# Inflammatory Myofibroblastic Tumor Presenting as a Partial Bowel Obstruction: A Case Report

**DOI:** 10.7759/cureus.36798

**Published:** 2023-03-28

**Authors:** Kolton Kaspar, Anthony Jackson, Christina M Hunt, Seth Williams, Donovan Trudeau, Ricardo Mohammed

**Affiliations:** 1 Department of Research, Alabama College of Osteopathic Medicine, Dothan, USA; 2 Department of General Surgery, Santa Rosa Medical Center, Milton, USA

**Keywords:** small-bowel intussusception, lead point, inflammatory myofibroblastic tumor (imt), whirl sign, small-bowel obstruction

## Abstract

Intussusception, or telescoping of the bowel, is a rare condition in the adult population that can lead to serious complications, such as obstruction or ischemia. Most cases of intussusception are idiopathic and present with a pathognomonic “target sign” on imaging. Rarely, in adults, intussusceptions can be found with lead points, some of which may be neoplastic. Treatments for intussusception include air enemas or surgical intervention if enemas are unsuccessful in resolving the telescoped bowel. This case report discusses an atypical presentation of intussusception in an adult female with a “whirlpool sign” on imaging rather than the typical “target sign.” She was found to have incorporation of mesenteric fat into telescoping bowel causing edema and partial bowel obstruction. The affected bowel was removed laparoscopically, and an end-to-end anastomosis was formed. Pathology of the resected bowel revealed a non-immunoreactive inflammatory myofibroblastic neoplasm as the lead point. Most inflammatory myofibroblastic tumors stain positive for desmin, smooth muscle actin, and anaplastic lymphoma kinase (ALK), whereas this patient was non-immunoreactive. The patient tolerated surgery well and is now pain-free with normal gastrointestinal function. This case report hopes to heighten awareness of atypical presentations of intussusceptions, the use of imaging to help aid in uncertain diagnoses, and the appropriate surgical treatment for symptomatic patients.

## Introduction

Intussusception occurs when a proximal segment of bowel telescopes into a distal segment. It accounts for 1% of small bowel obstructions in adults, and currently 95% of diagnosed cases are in the pediatric population. The underlying etiology is often idiopathic in the pediatric population; however, a lead point is found in most cases of intussusception in adults [1].

Inflammatory myofibroblastic tumors are uncommon neoplasms that, when found, are typically seen in the adolescent and pediatric populations. These neoplasms are often localized to the abdominal cavity within the omentum and mesentery [2]. We present a case of intussusception in an adult female secondary to an inflammatory myofibroblastic neoplasm that was managed surgically.

## Case presentation

A 60-year-old Caucasian female presented to the emergency department with the chief complaint of abdominal pain for the past four days. The pain was described as sharp, constant, and worsening over the past 12 hours. The pain was periumbilical with radiation to her lumbar region. There was anorexia, nausea, and two episodes of non-bilious emesis. The patient reports a bowel movement earlier in the day and frequent eructation. Past medical history included gastritis, hypertension, and GERD. Prior surgical history was notable for tubal ligation. The patient reported a partial small bowel obstruction that was managed medically seven months prior. Current medications include simethicone, omeprazole, carvedilol, and verapamil daily.

**Figure 1:**
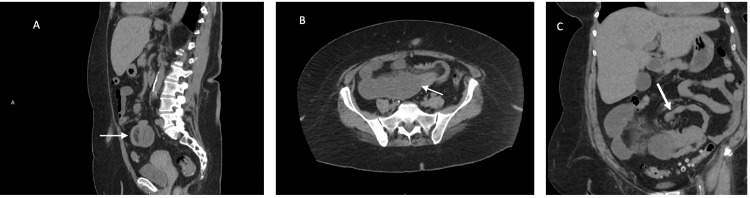
Non-contrast abdominal CT of the patient. The images show (A) sagittal view significant for an incomplete target sign, (B) transverse view of the intussusception, and (C) in the coronal view the whirl sign became evident.

Physical examination revealed a middle-aged obese female with voluntary guarding. The laboratory evaluation was unremarkable. Abdominal CT without contrast revealed dilated small bowel in the mid-abdomen with areas of narrowing (Figures 1A-1C). The radiologist read it as questionable for a “partial intussusception versus partial closed-loop bowel obstruction.” Inflammatory changes were noted in the mesentery.

**Figure 2:**
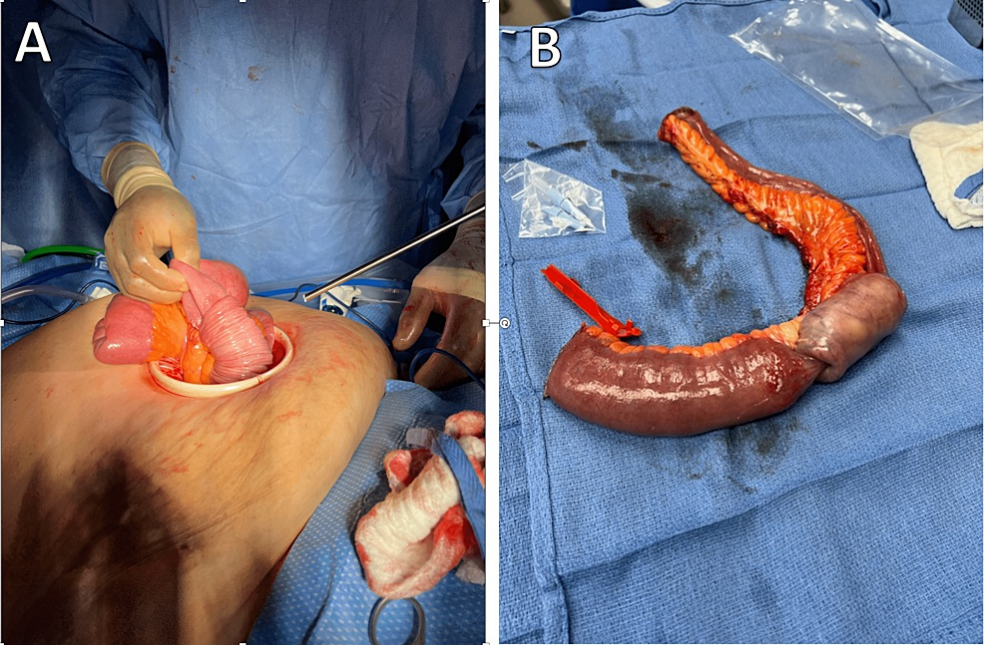
Jejunoileal intussusception being extracted through a hand assist port (A) and excised segment of bowel (B).

After discussing risks, benefits, and alternatives with the patient, a diagnostic laparoscopy with possible conversion to open laparotomy was scheduled. Visualization of the small bowel revealed an intussusception in the distal jejunum extending into the proximal ileum (Figures 2A, 2B). A hand-assisted port was placed. The affected bowel was excised, and an end-to-end anastomosis was created.

The removed bowel measured 34 cm and was sent for pathology. The Pathology report revealed a 6.5 cm mass that demonstrated spindle cell proliferation with mixed inflammatory cells. The neoplasm was non-reactive to the following: CD117, DOG1, STAT6, beta-catenin, anaplastic lymphoma kinase-1 (ALK-1), desmin, muscle actin, S-100, and CD34. The diagnosis is an inflammatory myofibroblastic tumor.

## Discussion

Intussusception is a common cause of small bowel obstructions in the pediatric population. However, it is rarely seen in adults, with the diagnosis of intussusception being made in 1% of patients with an underlying small bowel obstruction [1]. The diagnosis of intussusception may be challenging due to its variable presentation in the adult population [3]. In contrast, the diagnosis in the pediatric population appears to be more straightforward. The typical triad of symptoms seen is abdominal pain, bloody stool, and vomiting [4]. In adults, abdominal pain was the most reported symptom.

Computerized tomography (CT) and ultrasound (US) are commonly used to aid in the diagnosis. “Target sign” is pathognomonic for intussusception on either US or CT [5]. In this case, the telescoping segment of the small bowel incorporated mesenteric fat. The resulting presentation was a whirlpool sign, which is associated with bowel volvulus [6]. The final decision to operate was made based on the presence of mesenteric edema and patient symptomology.

Most cases of intussusception in adults are due to a lead point. The lead point can be benign or malignant. The lead point in this patient was an inflammatory myofibroblastic tumor (IMT). A total of 80-90% of cases of inflammatory myofibroblastic tumors stain positive for desmin and smooth muscle actin [2]. Other common staining features of IMT include vimentin and cytokeratin, and ALK if malignant [2,7]. In this case, the patient discussed was challenging to diagnose due to the abnormal staining features. The diagnosis of IMT was made as a diagnosis of exclusion.

This was a challenging case that was confounded by multiple factors. First, the patient was seen by providers at different facilities with non-communicating electronic medical records. Second, the most common radiographic findings associated with intussusception were obscured due to the incorporation of mesenteric fat. Finally, the neoplasm fit the description of an inflammatory myofibroblastic tumor but failed to stain positive for the most common immunohistochemical markers.

## Conclusions

Intussusception is a challenging diagnosis based on its varied presentation. This case is meant to bring attention to a unique variation. The case here was distinctive because the patient was outside of the typical age range for the diagnosis of both intussusception and IMT. The patient experienced two episodes over one year before receiving definitive care. Radiographically, a whirlpool sign was found instead of the classic target sign. Finally, the lead point was determined to be a non-immunoreactive inflammatory myofibroblastic neoplasm.

## References

[1] Panzera F, Di Venere B, Rizzi M (2021). Bowel intussusception in adult: prevalence, diagnostic tools and therapy. World J Methodol.

[2] Gleason BC, Hornick JL (2008). Inflammatory myofibroblastic tumours: where are we now?. J Clin Pathol.

[3] Azar T, Berger DL (1997). Adult intussusception. Ann Surg.

[4] Macdonald IA, Beattie TF (1995). Intussusception presenting to a paediatric accident and emergency department. J Accid Emerg Med.

[5] Gayer G, Zissin R, Apter S, Papa M, Hertz M (2002). Pictorial review: adult intussusception--a CT diagnosis. Br J Radiol.

[6] Singh D, Chawla A (2016). The "abdominal whirlpool" sign. Abdom Radiol (NY).

[7] Sastre-Garau X, Couturier J, Derré J, Aurias A, Klijanienko J, Lagacé R (2002). Inflammatory myofibroblastic tumour (inflammatory pseudotumour) of the breast. Clinicopathological and genetic analysis of a case with evidence for clonality. J Pathol.

